# Differences in beliefs about mood between people with and without bipolar disorder

**DOI:** 10.1002/cpp.2391

**Published:** 2019-08-14

**Authors:** Heather Robinson, Steven Jones, Thomas Fanshawe, Fiona Lobban

**Affiliations:** ^1^ Spectrum Centre for Mental Health Research, Department of Health Research Lancaster University Lancaster UK; ^2^ Nuffield Department of Primary Care Health Sciences University of Oxford Oxford UK

**Keywords:** affect, beliefs, bipolar, experience sampling, mood

## Abstract

Psychological models of bipolar disorder (BD), such as the self‐regulation model (SRM; Leventhal, Nerenz, & Steele, 1984), highlight the crucial role of beliefs about mood in relapse vulnerability. To date, no studies have directly compared these beliefs between people with and without BD. Based on the SRM, the current research examined beliefs about mood in people with and without BD and explored the impact of current affect on these beliefs. Fifty euthymic people with a diagnosis of BD and 50 controls were recruited through an online screening study, clinical services, and support organizations. Experience sampling methodology (ESM) was used to assess beliefs (according to the Brief Illness Perceptions Questionnaire; Broadbent, Petrie, Main, & Weinman, 2006) across a typical week of everyday life. Data were analysed using multilevel modelling. Forty‐two people with a diagnosis of BD and 50 controls were included in the analyses. Results indicated that the BD group reported less control over mood, a shorter duration of mood, and less understanding of mood and were more likely to report the cause of depressive symptoms as something internal, compared with controls. When controlling for current affect, the BD group also reported more positive consequences, made more internal attributions for hypomanic symptoms, and reported less concern about mood, compared with controls. Findings suggest important differences in beliefs about mood between people with and without BD that are not the result of current affect. These beliefs may be particularly important in understanding underlying vulnerability to future relapse into depression and/or mania.

**Key Practitioner Message**

- Specific beliefs about mood differ between people with and without bipolar disorder (BD), a subset of which are unaffected by changes in current affect and may therefore represent a core belief set in BD.
- When controlling for current affect, people with a diagnosis of BD reported beliefs that may affect escalation to hypomania.
- Psychoeducational and cognitive‐behavioural approaches should include techniques to address internal appraisal mechanisms for mood changes. Recovery‐focussed therapy could provide a way to do this.
- Psychological interventions should acknowledge the positive aspects of mood escalation and the possible risk factors and genuinely encompass both. Ambivalence to treatment is likely where this does not happen and the focus is only on risk.

## 1 INTRODUCTION

Everyone experiences changes in mood. Psychological models of bipolar disorder (BD) attempt to explain why some people are able to regulate these changes, but for others, mood change can amplify into an episode of depression or mania. Such models highlight the crucial role of beliefs about current mood in relapse vulnerability.

The importance of beliefs about circadian rhythm disruption in relapse vulnerability is highlighted in the Schematic, Propositional, Analogical and Associative Representational Systems model for BD (SPAARS; Jones, 2001). Jones (2001) applied the original SPAARS model (Power & Dalgleish, 1997) to mania and depression in BD, suggesting that the analogical system (which incorporates olfactory, auditory, gustatory, visual, proprioceptive, and tactile modalities) can become disrupted through circadian rhythm changes (e.g., insomnia, jetlag, drug/alcohol use, and daylight changes) and that internal appraisals of this disruption (held at the schematic, propositional, and associative levels) may increase vulnerability to BD onset and recurrence (Healy & Williams, 1989; Jones, 2001).

Based on this model, the Hypomanic Interpretations Questionnaire (HIQ; Jones, Mansell, & Waller, 2006) and the Interpretations of Depression Questionnaire (IDQ; Jones & Day, 2008) were developed to measure the tendency for people to believe that hypomanic or depressive symptoms respectively were linked to something internal/related to the self rather than external factors. For example, “If I felt in high spirits and full of energy, I would probably think it was because I am a talented person with lots to offer” (self‐dispositional hypomanic appraisal) or “If I felt down on myself, I would probably think it was because I am a bad person” (self‐dispositional depressive appraisal). Previous studies have found that people with BD score significantly higher on the HIQ and IDQ compared with controls (Banks, Lobban, Fanshawe, & Jones, 2016; Jones et al., 2006; Mansell & Jones, 2006) and that this may be a vulnerability factor in BD (Jones & Day, 2008; Jones, Hare, & Evershed, 2005).

The importance of beliefs about changes in internal state (including mood state) is also highlighted in the Integrative Cognitive Model (ICM; Mansell, Morrison, Reid, Lowens, & Tai, 2007). The ICM suggests that beliefs that a change in mood has extreme personal meaning provoke exaggerated efforts to control that mood change. Such efforts exacerbate the initial mood change, resulting in a vicious cycle of mood intensification.

The Hypomanic Attitudes and Positive Predictions Inventory (HAPPI; Mansell, 2006) was developed to measure the extreme, personal positive and negative beliefs about internal states central to ICM. A number of studies have reported an association between scores on the HAPPI and BD‐related experiences in nonclinical samples (Dodd, Mansell, Beck, & Tai, 2013; Dodd, Mansell, Bentall, & Tai, 2011; Mansell, Rigby, Tai, & Lowe, 2008). Preliminary evidence has been found for the predictive validity of the HAPPI for BD symptoms in a clinical sample (Dodd, Mansell, Morrison, & Tai, 2011), and people with BD have been found to score higher on the HAPPI than matched nonclinical and unipolar samples, while controlling for current symptoms (e.g., Alatiq, Crane, Williams, & Goodwin, 2010; Mansell, 2006; Mansell et al., 2011; Mansell & Jones, 2006).

The self‐regulation model (SRM; Leventhal et al., 1984) is a theoretical framework that attempts to explain how people use coping behaviours to try to bring their current health state in line with their desired health state. The beliefs people hold about their illness experiences guide how coping strategies are employed, which subsequently influences outcome. The SRM has proved to be helpful for understanding illness beliefs and their association with outcomes in physical health, providing useful information to inform treatment (Hagger & Orbell, 2003), and has since been applied to mental health problems (Baines & Wittkowski, 2012; Lobban, Barrowclough, & Jones, 2004; Peay, Rosenstein, & Biesecker, 2013). The Brief Illness Perception Questionnaire (BIPQ; Broadbent et al., 2006) is a measure of the illness beliefs proposed by the SRM. In a recent cross‐sectional study (Dodd, Mezes, Lobban, & Jones, 2017), negative beliefs about mood swings were shown to be associated with poorer personal recovery in BD. However, only one study to date has used the BIPQ to explore the impact of beliefs about mood on outcomes over time in BD. In a study of 91 BD participants, Lobban, Solis‐Trapala, Tyler, Chandler, and Morriss (2013) found that illness beliefs had important effects on symptomatic outcomes over a 24‐week period. Specifically, higher perceived consequences, more symptoms experienced (identity), and greater concern about mood were predictive of time to relapse over 24 weeks. Additionally, higher perceived consequences and lower perceived effort to get well were associated with higher weekly depressive symptom scores after controlling for baseline depression, medication, and number of previous episodes. Although this study indicates that beliefs about mood may be a vulnerability factor for relapse, it is not clear whether these beliefs could be a vulnerability factor specifically for BD or mood fluctuations in general. Furthermore, despite beliefs about consequences being a strong predictor of outcome in this, and numerous other non‐BD studies (Broadbent et al., 2015), the extent to which positive and negative consequences separately predicted outcome was not examined. Therefore, we cannot determine whether beliefs about positive and negative consequences lead to different outcomes.

All of these models hypothesize that beliefs about mood may be a vulnerability factor for BD. We aimed to explore whether such beliefs have a specific role in determining mood changes in people with BD compared with controls and therefore may be a vulnerability factor for extreme fluctuations in mood. We chose to use the SRM as our theoretical framework as this is inherently transdiagnostic in its development and application and therefore can be applied to people in both groups of the study.

In order to examine differences in beliefs between people with and without BD in real time, during a typical week of everyday life and in the natural environment, we employed Experience Sampling Methodology (ESM). People with BD are more likely to be in lower affective state and show stronger fluctuations in affect, than those without BD, even when not in episode (Judd et al., 2002; Judd et al., 2003; Knowles et al., 2007). Therefore, to test whether differences in current affect impact on underlying beliefs about mood, we assessed beliefs with and without controlling for current affect. Our aims were

1. To identify differences in beliefs about mood between people with and without BD across a typical week of everyday life, using ESM.
2. To examine whether current affect can explain reported differences in beliefs by controlling for current affect in‐group comparisons.

## 2 METHODS

This study was conducted in line with the Declaration of Helsinki. The study was approved by a UK local National Health Service (NHS) ethics committee (REC ref: 10/H1015/76), and additionally independently peer reviewed and adopted by the Mental Health Research Network in England (MHRN ref: 59258). All participants provided written informed consent.

### 2.1 Sample

A convenience sample of 50 people with a diagnosis of BD and 50 controls were recruited through an online screening study advertised using online adverts on internet forums and media sites, and poster and newsletters circulated around universities across the North West of England (Banks et al., 2016). Additional participants were recruited directly through local clinical services and support organizations. BD I or II was confirmed using Structured Clinical Interview for DSM‐IV (SCID; First, Spitzer, Gibbon, & Williams, 1997). Control participants were required to score < 0.5 standard deviations above the sample mean on the Hypomanic Personality Scale (HPS; Eckblad & Chapman, 1986), to exclude people at high behavioural risk for BD (Kwapil et al., 2000). Additional eligibility criteria for both groups included

- Aged 18 years old or over.
- No current manic, hypomanic, mixed affective, or major depressive episode currently or within the 4 weeks prior to baseline assessment assessed by SCID‐Life (First et al., 1997) scores.
- No physical brain injury.
- No current suicide plans or high suicide intent.
- Able and willing to give written informed consent to the study.
- Able to communicate in written and oral English to a sufficient level to allow the participant to complete the measures.
- Not a night shift worker.

### 2.2 Experience sampling methodology (ESM)

ESM refers to a set of empirical methods that allow participants to respond to questions within the context of their daily lives, following a specific event or at a specific time (often signalled by a watch or mobile device; Christensen, Barrett, Bliss‐Moreau, Lebo, & Kaschub, 2003). Thoughts, feelings, and experiences are captured in detail (through repeated assessments often using a paper diary or mobile device), as they occur (momentary assessments of current state), and in the context in which they occur (increasing ecological validity). ESM is a well‐established approach for investigating processes in mental health research. ESM has been employed to investigate cross‐sectional (Myin‐Germeys, Delespaul, & van Os, 2003) and temporal (Pavlickova et al., 2013) associations between variables in BD, as well as biological markers (Havermans, Nicolson, Berkhof, & deVries, 2011) and as a technique for intervention (Depp et al., 2010). However, ESM has not yet been used to examine the types of beliefs measured by the BIPQ, which has generally relied on cross sectional self‐report and therefore does not address the dynamic nature of BD and neglects contextually and temporally sensitive information such as changes in current affect. By using multiple, frequent assessments of momentary phenomena, ESM can provide an ecologically valid, real‐time method for examining the inherently dynamic nature of BD that traditional assessments fail to capture. As with the majority of ESM studies using BD samples (e.g., Fulford, Johnson, Llabre, & Carver, 2010; Havermans et al., 2011; Havermans, Nicolson, Berkhof, & deVries, 2010; Havermans, Nicolson, & Devries, 2007; Knowles et al., 2007; Myin‐Germeys et al., 2003; Pavlickova et al., 2013), we used momentary paper diary assessments in real time triggered by a watch/mobile phone signal. To ensure accurate completion of diaries, participants recorded the time at which they completed each diary entry and were instructed to complete the diary as soon as possible following an alert (within no more than 10 min). Where a response was missed, participants were asked to provide a reason and to wait until the next alert to respond. The number of diary items selected was consistent with good practice in ESM research (Palmier‐Claus et al., 2011). See Section 2.4 for more detail.

### 2.3 Measures

**Figure 1:**
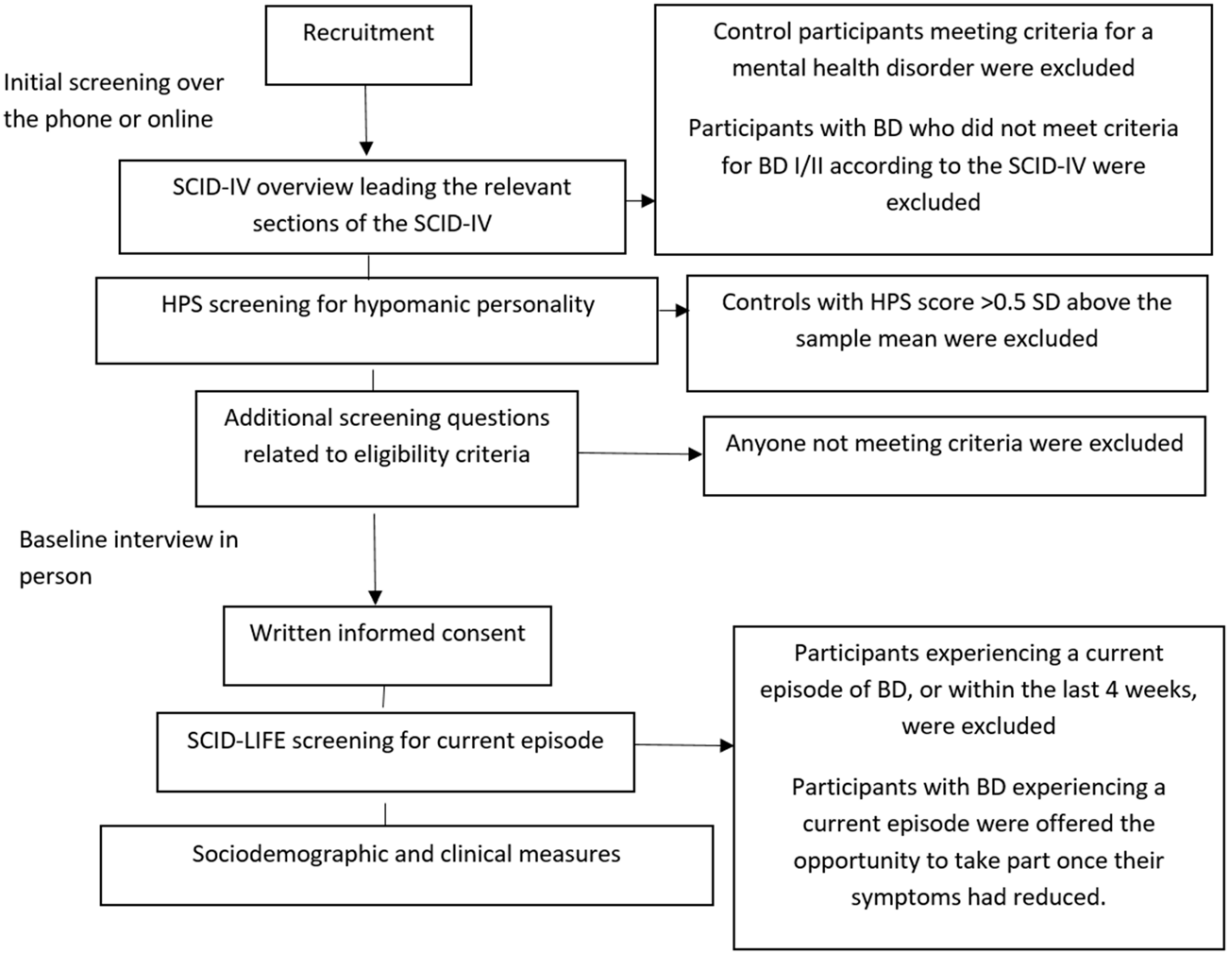
Flow chart of the screening process

Screening measures (see Figure 1)

The *HPS* (Eckblad & Chapman, 1986) was used to screen controls (participants who scored < 0.5 standard deviations above the sample mean on the HPS).

The *SCID* (First et al., 1997) was used to confirm that the clinical sample met research diagnostic criteria for BD I or II to verify any additional DSM‐IV Axis I psychological disorders and to screen control participants. SCID interviews were administered over the telephone by a trained researcher to allow participants across the North of England to participate.

The *SCID Life* (First et al., 1997) was administered at baseline to assess participants' mood during the previous 4 weeks. It incorporates the Hamilton Depression Rating Grid (Williams et al., 2008) and the Bech‐Rafaelsen Mania Scale (Bech, Rafaelsen, Kramp, & Bolwig, 1978), which also provided measures of depressive or manic symptoms at baseline.

ESM Diary (available on request)

*Affect assessment*: Momentary affect assessment diary items were drawn from items used in previous ESM research assessing positive and negative affect in clinical samples (e.g., Havermans et al., 2010) and validated measures of affect (e.g., Positive and Negative Affect Schedule (Watson, Clark, & Tellegen, 1988) and the Internal State Scale (Bauer et al., 1991)). Item ratings for “cheerful,” “energetic,” and “confident” were averaged to assess positive affect, and “bad about myself,” “down,” and “guilty” were averaged to form a negative affect scale. All were rated on a 7‐point Likert scale (1 to 7 [*very*]).

The *BIPQ* (Broadbent et al., 2006) was used to measure beliefs about mood. The BIPQ was adapted for use with a control, as well as clinical, sample by omitting the treatment control dimension (as this is not relevant to the former group) and emotional representation (due to its overlap with our focus of interest on mood; see Appendix A). The following dimensions were included: identity (current affect), beliefs about consequences (positive and negative), personal control, concern, comprehensibility, and duration (time line). Beliefs about the cause of current mood were assessed using single items from the HIQ (Jones et al., 2006; Item “If I felt in high spirits and full of energy, I would probably think it was because I'm a talented person with lots to offer”) and the IDQ (Jones & Day, 2008; “If I felt down on myself, I would probably think it was because I am a bad person, even towards myself”*)* to allow multilevel modelling analysis. These were selected based on the items with the highest loading in principal components analyses of these positive and negative appraisal measures.

### 2.4 Procedure

Participants were screened (using the SCID overview leading to relevant sections of the SCID) for primary BD diagnosis (or lack of) and completed the HPS online. Following further screening questions over the phone to verify eligibility, participants were invited to take part in a baseline interview in person. Participants gave written informed consent and were screened using the SCID Life. Participants with BD who could not be included due to scores above the threshold (SCID Life score > 4 indicating more than normal mood symptoms) were offered the opportunity to take part once their symptoms had reduced. Participants then completed sociodemographic and clinical measures (listed in Table 1) prior to a briefing session to explain use of the diaries. Participants were provided with seven A5 ESM diaries (one for each day) and an information book detailing study procedure.

**Table 1:** Demographic characteristics and clinical features of control and bipolar disorder groups

Descriptive	Control eligible (*n* = 50)	BD eligible (*n* = 42)	Test statistic	df	*p* value
Gender ratio (M/F)	10/40	16/26	X^2^ = 3.69	1	.06
Age, mean (SD)	37.64 (9.91)	43.95 (12.74)	t = −2.67	90	.01
Highest level of education, *n* (%)			X^2^ = 13.92	2	< .001
Secondary	2 (4%)	11 (26%)			
Further	8 (16%)	12 (29%)			
Higher	40 (80%)	19 (45%)			
Employment status, *n* (%)			X^2^ = 24.78	1	< .001
Working (paid PT/FT)	43 (86%)	15 (36%)			
Not working	7 (14%)	27 (64%)			
Marital status, *n* (%)			X^2^ = 6.53	2	.04
Single	12 (24%)	15 (36%)			
Married/cohabiting	34 (68%)	18 (43%)			
Separated/divorced/widow	4 (8%)	9 (21%)			
Living arrangements, *n* (%)			X^2^ = 7.83	2	.02
Partner with/without others	33 (66%)	18 (43%)			
Alone	6 (12%)	15 (36%)			
Other	11 (22%)	9 (21%)			
Nationality, *n* (%)			X^2^ = 1.14	1	.29
British	48 (96%)	38 (90%)			
Other	2 (4%)	4 (10%)			
BD type, I/II	‐	32/10			
Psychological treatment for mood (*n*, %)					
No treatment	‐	16 (38%)			
Current treatment	‐	7 (17%)			
Past treatment	‐	15 (36%)			
Information missing	‐	4 (10%)			
No. depressive episodes, *n* (%)					
0	‐	2 (5%)			
1–6	‐	14 (33%)			
7–11	‐	6 (14%)			
12–29	‐	14 (33%)			
30+	‐	6 (14%)			
No. manic/hypomanic episodes, *n* (%)					
1–6	‐	18 (43%)			
7–11	‐	7 (17%)			
12–29	‐	11 (26%)			
30+	‐	6 (14%)			
No. hospitalizations, n (%)					
0	‐	15 (36%)			
1–6	‐	22 (52%)			
7–11	‐	2 (5%)			
12–29	‐	3 (7%)			
Medication					
Monotherapy	‐	10 (24%)			
Combined therapy	‐	30 (71%)			
Antidepressant	‐	16			
Lithium	‐	10			
Valproate	‐	10			
Carbamazepine	‐	3			
Lamotrigine	‐	6			
Benzodiazepines/hypnotics	‐	10			
Antipsychotics	‐	30			
Meds for physical problems	‐	17			

Abbreviations: BD = bipolar disorder. FT = full time. PT = part time. SD = standard deviation.

Participants were alerted to the need to fill in the ESM diary by either text alerts from a mobile phone or beeps from an Ironman Data Link watch (five BD and five control participants chose to use a watch and the remaining participants used mobile phones). Text or beep alerts prompted participants to complete the same set of diary questions (see above) 10 times a day for 7 days. All alerts were programmed to occur between 7:45 a.m. and 10:15 p.m., at pseudo‐random intervals. The minimum gap between alerts was 24 min and the maximum was 159 min, with an average gap of 90 min.

Participants who completed fewer than 30% of alerts within 10 min of the alert were excluded, consistent with a minimum threshold of 20 completed reports (Palmier‐Claus et al., 2011). Again, consistent with good practice for ESM, participants were contacted by phone during the study week to provide support, increase motivation, and arrange a final appointment (Palmier‐Claus et al., 2011). During this appointment, the researcher collected the study equipment, debriefed the participant, and gave a £10 “thank you” for their involvement.

### 2.5 Statistical analysis

Data were analysed in R (R Development Core Team, 2011) using multilevel modelling (MLM), which is appropriate for the nested structure of the ESM data (participants' responses nested within day, within participant). An MLM was formulated in which participant‐level variance was estimated to account for interindividual variability. The model was adjusted for diurnal and hourly variations in responses by including day‐of‐week and time‐of‐day terms. To account for correlation between measurements that depend on the time of measurement, a time‐since‐first‐response correlation term was modelled. Autocorrelation was also modelled because observations from a participant that were closer in time were more likely to be more similar than observations further apart. To estimate effects of current affect on group differences in beliefs about mood (Aim 2), the general MLM framework was adjusted for positive affect (PA) and negative affect (NA) simultaneously using two separate parameters for each affect type. Statistical significance was interpreted as *p* ≤ .05. The Bonferroni correction for multiple tests has been criticized for being overly conservative (Hochberg & Benjamini, 1990); to ensure a low false positive rate (< 5%), we used the more powerful false discovery rate (FDR; Glickman, Rao, & Schultz, 2014).

As age and gender did not improve the goodness‐of‐fit according to the Akaike information criterion value, they were not included in the final MLM. Only variables directly associated with the current hypotheses and those necessary for appropriate analysis of ESM data were included.

## 3 RESULTS

### 3.1 Participant sample

The final sample was 92 adults with either BD (*n* = 42) or no history of mental health problems (control; *n* = 50). Eight participants with BD were excluded because they completed fewer than 30% of ESM alerts within 10 min of the signal.

The demographic and clinical characteristics of the sample are displayed in Table 1. Both groups were predominantly female and White British. BD participants were older, had less experience of higher education, were less likely to be working, were more likely to be single or divorced, and were less likely to be living with a partner than controls.

The majority of the BD group had a diagnosis of BDI with an established course of BD: Over half had experienced at least seven episodes of mania and/or depression and between 1 and 29 hospitalizations. The majority of the BD group had never received psychological treatment for mood (38%) or had received past treatment (36%). Seventeen per cent of BD group were receiving current psychological treatment for mood and the majority (95%) were taking some form of medication (none of the control group were taking prescription medication). Twenty four per cent of the BD sample were prescribed monotherapy (lithium, *n* = 2; carbamazepine, *n* = 2; and antipsychotics, *n* = 6). Seventy one per cent were prescribed 2–6 medications in different combinations: antidepressants (*n* = 16), lithium (*n* = 8), valproate (*n* = 10), carbamazepine (*n* = 1), lamotrigine (*n* = 6), benzodiazepines (*n* = 10), antipsychotics (*n* = 24), and medication for physical problems (*n* = 17).

### 3.2 ESM results

There were 4,197 eligible responses (within 10 min of the alert) in total. Sixty eight per cent of alerts sent to controls and 55% of alerts sent to BD were eligible. Fifteen per cent of ineligible control alerts were completed after 10 min (23% for BD alerts); the remainder were missed completely. Explanations for missing data were provided in 66% of cases for controls and 43% for BD. Controls missed alerts most often due to work or study, whereas those with BD reported most commonly being asleep or resting.

Aim 1 Identify any differences in beliefs about mood between people with and without BD across a typical week of everyday life

The BD group reported significantly less personal control, more concern, a significantly shorter expected duration of current mood, less understanding of mood, and more likely to attribute the cause of depressive symptoms to something internal compared with controls (see Table 2). These group differences remained significant after the FDR correction.

Aim 2 Examine the effect of current affect on differences in beliefs

**Table 2:** Differences in beliefs between BD and control

Belief	BD mean (SD)	Control mean (SD)	MD	95% CI (lower, upper)	Group *p* value
Personal control	5.91 (2.65)	7.21 (1.91)	−1.29	−2.04, −0.55	<.001*
Concern	2.64 (2.14)	1.64 (1.36)	0.97	0.46, 1.49	<.001*
Time line	5.23 (2.42)	6.32 (1.94)	−0.92	−1.60, −0.23	<.01*
Comprehensibility	6.71 (2.26)	7.92 (1.73)	−1.04	−1.72, −0.37	<.01*
Consequences^a^	0.27 (1.74)	0.70 (1.45)	−0.39	−0.80, 0.03	.07
Cause: HIQ‐H	1.89 (0.81)	1.76 (0.75)	0.15	−0.12, 0.41	.28
Cause: IDQ‐D	1.33 (0.65)	1.03 (0.20)	0.30	0.17, 0.43	<.001*

Consequences: the MLM was performed taking account of the direction of the effect (positive/negative).

Abbreviations: CI = confidence interval. MD = mean difference. SD = standard deviation.

* Remained significant after FDR correction.

There were statistically significant differences between groups in both positive (*MD* = −0.52, *p* = .01) and negative (*MD* = 0.79, *p* ≤ .001) affect. PA and NA were moderately negatively correlated with each other (*r* = −0.43, *p* < .001).

Table 3 displays the effect of group (“Group difference,” Columns 1 and 2), PA (“Positive affect,” Columns 3 and 4), and NA (“Negative affect,” Columns 5 and 6) on each type of belief. When current affect was controlled for, the BD group still reported less control over mood, a shorter expected duration of mood, less understanding of mood and were more likely to attribute the cause of depressive symptoms to something internal compared with controls. In addition, controlling for current affect impacted on group differences in beliefs about consequences and cause: BD participants reported significantly more positive consequences associated with mood and were more likely to attribute the cause of hypomanic symptoms to something internal compared with controls. These group differences remained significant after the FDR correction. There were no longer statistically significant group differences in concern.

**Table 3:** Difference in beliefs between BD and control when current affect was controlled

Beliefs	Group difference		Positive affect		Negative affect	
	MD (95 % CI)	Group *p* value	PA MD (95% CI)	PA *p* value	NA MD (95% CI)	NA *p* value
Personal control	−0.74 (−1.34, −0.14)	.02*	0.69 (0.64, 0.75)	<.001	−0.26 (−0.33, −0.19)	<.001
Concern	0.15 (−0.21, 0.50)	.42	−0.23 (−0.27, −0.19)	<.001	0.91 (0.85, 0.97)	<.001
Time line	−0.82 (−1.43, −0.21)	.01*	0.61 (0.56, 0.67)	<.001	0.28 (0.21, 0.36)	<.001
Comprehension	−0.85 (−1.47, −0.22)	<.01*	0.33 (0.28, 0.37)	<.001	−0.08 (−0.15, −0.02)	.01
Consequences^a^	0.39 (0.08, 0.71)	.02*	0.82 (0.79, 0.86)	<.001	−0.45 (−0.50, −0.40)	<.001
Cause: HIQ‐H	0.27 (0.03, 0.51)	.03*	0.19 (0.17, 0.20)	<.001	−0.06 (−0.08, −0.03)	<.001
Cause: IDQ‐D	0.12 (0.04, 0.21)	.01*	−0.05 (−0.06, −0.04)	<.001	0.21 (0.19, 0.22)	<.001

a Consequences: the multi‐level modelling was performed taking account of the direction of the effect (positive/negative)

Abbreviations: HIQ = Hypomanic Interpretations Questionnaire. IDQ = Interpretations of Depression Questionnaire. MD = mean difference. NA = negative affect. PA = positive affect.

* Remained significant after FDR correction

In both groups, higher PA and lower NA were associated with reports of more personal control, understanding, positive consequences, and internal attributions for hypomanic symptoms. Lower PA and higher NA were associated with more concern and internal attributions for depressive symptoms. Both higher PA and higher NA were associated with beliefs about longer duration of mood.

## 4 DISCUSSION

This study explored differences in beliefs about mood between people with and without BD across 7 days.

During a typical week, people with BD reported significantly less personal control, more concern, a shorter duration of current mood, and less understanding of mood and were more likely to attribute the cause of depressive symptoms to something internal, compared with people without BD. All of these differences, apart from greater concern, remained when current affect was controlled for and are therefore unlikely to be due to reporting biases associated with current affect. These beliefs may be particularly important in BD because they may distinguish those who go on to experience worse clinical outcomes. Indeed, the importance of beliefs about control has been highlighted previously (e.g., Crowe et al., 2012; Mansell et al., 2007; Proudfoot et al., 2012). In a recent review and meta‐analysis of the BIPQ, Broadbent et al. (2015) highlighted the importance of beliefs about personal control for outcomes in a range of psychological disorders, including BD.

The current findings regarding control have important implications both for psychological therapy and for the wider treatment of people with BD. Although perceptions of a short duration of current mood and lack of control over current mood in BD may be accurate for some people given that BD is associated with increased mood variability even in remission (Knowles et al., 2007), psychological interventions should aim to promote self‐management techniques, allowing people with BD to take control over their lives and emotions. Where people are given a message that their disorder is purely driven by biological and genetic factors, a sense of personal control is likely to be diminished. Psychosocial models recognize that the ways individuals respond to initial mood changes affect how they develop and are more likely to enhance a sense of personal control and personal understanding of mood experiences. The importance of delivering a balanced message of this type has been recognized by National Institute for Health and Clinical Excellence (2014).

A possible explanation for why people with BD struggle to control their mood is that they cannot make sense of what is happening. Consistent with studies of illness comprehension in other mental health disorders (e.g., Godoy‐Izquierdo, Lo'pez‐Chicheri, Lo'pez‐Torrecillas, Ve'lez, & Godoy, 2007; Higbed & Fox, 2010), current findings suggest that people with BD lack a coherent model regarding their mood, regardless of current affect. This is despite the development of formulations often being a significant part of clinical intervention (52% of the BD group had current or had received past, psychological treatment for mood). Furthermore, a coherent understanding of mood experiences is related to sense of personal recovery in BD (Jones et al., 2015; Morrison et al., 2016).

Whereas beliefs about control, duration, understanding, and self‐dispositional depressive appraisals were not affect dependent, beliefs about positive consequences and self‐dispositional hypomanic appraisals in BD were associated with PA and therefore were only revealed when low affect in BD was controlled for in analyses. Similarly, beliefs about concern were influenced by current affect. When current affect was not controlled, consistent with previous research (Lobban et al., 2013), we found increased concern in people with BD. However, concern was associated with higher NA, which was elevated in the BD group. When this was controlled for, group differences in concern disappeared. As mood increases, these less catastrophizing, more positive beliefs may become more apparent and could influence escalation into hypomania. The challenge for interventions is to acknowledge positive aspects of BD and possible risks associated with mood escalation in order to genuinely encompass both aspects into therapy. Positive aspects of BD are highly valued by some people, and the chance to talk about them is welcomed (Lobban, Taylor, Murray, & Jones, 2012). Ambivalence towards treatment is likely where the focus is only on risk, ignoring the positive aspects of BD. Indeed, there is evidence that positive illness models enhanced recovery (Dodd et al., 2017).

Taken together, current results have important clinical implications. Structured psychological interventions are effective in reducing relapse risk in BD (Oud et al., 2016). These generally share common elements of providing information about BD, improving cognitive and behavioural coping strategies and developing action plans to address relapse signs (Miklowitz, Goodwin, Bauer, & Geddes, 2008). Although it is clear that such approaches should be helpful in providing coherent illness models and improving sense of control, most of these approaches do not explicitly address internal appraisal mechanisms for mood changes or address the positive as well as negative aspects of mood. Enhancement of psychoeducational and cognitive behavioural approaches to include techniques to address these factors should, based on SRM theory, promote increased therapeutic effectiveness. Indeed, recovery‐focussed therapy includes these elements and does show promise in both reducing relapse risk and improving functional and recovery outcomes in BD (Jones et al., 2015).

### 4.1 Limitations and future directions

This study benefits from being theoretically driven by the SRM and diagnostically valid due to the completion of SCID diagnostic interviews. ESM allowed for multiple, momentary assessments during a typical week of everyday life, thus we collected a rich amount of data per participant and ensured high ecological validity. However, there are also limitations that should be considered in interpreting the findings.

First, as with most research studies, this study recruited a specific group of people with BD, generally through NHS secondary care. Therefore, caution should be paid to the generalizability of the current findings to the general population of people with BD. In addition to generalizability, the specificity of current findings should be tested in future research using a clinical control group matched on symptomatology to allow examination of beliefs specific to BD.

Second, groups differed on a number of demographic variables and clinical comorbidities. However, there is no evidence that these variables would explain differences in beliefs between people with and without BD. The majority of the BD group were taking some form of psychiatric medication, whereas the control group were not. It could be argued that medication could confound any group differences found by altering affect in the BD sample only; however, this was controlled for in the second set of analyses and so is very unlikely.

Finally, this research did not establish any causal links between beliefs and affect. Future research should explore whether changing these beliefs can impact on outcomes in BD, such as relapse.

In conclusion, these findings are consistent with previous literature and psychological models such as the SRM, which suggests that underlying beliefs about mood play a crucial role in mood fluctuations in BD. The current study builds on existing research literature by highlighting important differences in beliefs about mood between people with and without BD, which are not the result of current affect and which therefore may explain variation in outcome. Prospective studies are needed to examine the impact beliefs about mood have on important outcomes in BD, such as relapse.

## CONFLICT OF INTEREST

None.

## APPENDIX A

### Adapted BIPQ items for use in with clinical and control sample

**Table:**

My current mood…	**Negatively**			**Not affecting**			**Positively**			
• is affecting me at the moment	‐4	‐3	‐2	‐1	0	1	2	3	4	
My current mood…	**Not**									**Very**
• is controllable by me	1	2	3	4	5	6	7	8	9	10
• is causing me concern	1	2	3	4	5	6	7	8	9	10
• makes sense to me	1	2	3	4	5	6	7	8	9	10
• will continue for a long time	1	2	3	4	5	6	7	8	9	10

## References

[cpp2391-bib-0001] Alatiq, Y. , Crane, C. , Williams, J. M. G. , & Goodwin, G. M. (2010). Dysfunctional beliefs in bipolar disorder: Hypomanic vs. depressive attitudes. Journal of Affective Disorders, 122, 294–300. 10.1016/j.jad.2009.08.021 19773086

[cpp2391-bib-0002] Baines, T. , & Wittkowski, A. (2012). A systematic review of the literature exploring illness perceptions in mental health utilising the self‐regulation model. Journal of Clinical Psychology in Medical Settings, 20, 263–274. 10.1007/s10880-012-9337-9 23108509

[cpp2391-bib-0003] Banks, F. D. , Lobban, A. F. , Fanshawe, T. R. , & Jones, S. H. (2016). Associations between circadian rhythm instability, appraisal style and mood in bipolar disorder. Journal of Affective Disorders, 203, 166–175. 10.1016/j.jad.2016.05.075 27295373

[cpp2391-bib-0004] Bauer, M. , Crits‐Christoph, P. , Ball, W. , Dewees, E. , McAllister, T. , Alahi, P. , … Whybrow, P. (1991). Independent assessment of manic and depressive symptoms by self‐rating scale. Charateristics and implications for the study of mania. Archives of General Psychiatry, 48, 807–812. 10.1001/archpsyc.1991.01810330031005 1929771

[cpp2391-bib-0005] Bech, P. , Rafaelsen, O. J. , Kramp, P. , & Bolwig, T. G. (1978). The mania rating scale: Scale construction and inter‐observer agreement. Neuropharmacology, 17, 430–431. 10.1016/0028-3908(78)90022-9 673161

[cpp2391-bib-0006] Broadbent, E. , Petrie, K. , Main, J. , & Weinman, J. (2006). The brief illness perception questionnaire. Journal of Psychosomatic Research, 60, 631–637. 10.1016/j.jpsychores.2005.10.020 16731240

[cpp2391-bib-0007] Broadbent, E. , Wilkes, C. , Koschwanez, H. , Weinman, J. , Norton, S. , & Petrie, K. J. (2015). A systematic review and meta‐analysis of the Brief Illness Perception Questionnaire. Psychology & Health, 30, 1361–1385. 10.1080/08870446.2015.1070851 26181764

[cpp2391-bib-0008] Christensen, T. , Barrett, L. , Bliss‐Moreau, E. , Lebo, K. , & Kaschub, C. (2003). A practical guide to experience‐sampling procedures. Journal of Happiness Studies, 4, 53–78.

[cpp2391-bib-0009] Crowe, M. , Inder, M. , Carlyle, D. , Wilson, L. , Whitehead, L. , Panckhurst, A. , … Joyce, P. (2012). Feeling out of control: A qualitative analysis of the impact of bipolar disorder. Journal of Psychiatric and Mental Health Nursing, 19, 294–302. 10.1111/j.1365-2850.2011.01786.x 22074414

[cpp2391-bib-0012] Depp, C. , Mausbach, B. , Granholm, E. , Cardenas, V. , Ben‐Zeev, D. , Patterson, T. , … Jeste, D. (2010). Mobile interventions for severe mental illness: Design and preliminary data from three approaches. Journal of Nervous and Mental Disease, 198, 715–721. 10.1097/NMD.0b013e3181f49ea3 20921861 PMC3215591

[cpp2391-bib-0013] Dodd, A. L. , Mansell, W. , Beck, R. A. , & Tai, S. J. (2013). Self appraisals of internal states and risk of analogue bipolar symptoms in student samples: Evidence from standardised behavioural observations and a diary study. Cognitive Therapy and Research, 37, 981–995. 10.1007/s10608-013-9541-4

[cpp2391-bib-0014] Dodd, A. L. , Mansell, W. , Bentall, R. P. , & Tai, S. (2011). Do extreme beliefs about internal states predict mood swings in an analogue sample? Cognitive Therapy and Research, 35, 497–504. 10.1007/s10608-010-9342-y

[cpp2391-bib-0015] Dodd, A. L. , Mansell, W. , Morrison, A. P. , & Tai, S. (2011). Extreme appraisals of internal states and bipolar symptoms: The Hypomanic Attitudes and Positive Predictions Inventory. Psychological Assessment, 23, 635–645. 10.1037/a0022972 21500923

[cpp2391-bib-0016] Dodd, A. L. , Mezes, B. , Lobban, F. , & Jones, S. H. (2017). Psychological mechanisms and the ups and downs of personal recovery in bipolar disorder. British Journal of Clinical Psychology, 56, 310–328. 10.1111/bjc.12140 28543095

[cpp2391-bib-0017] Eckblad, M. , & Chapman, L. J. (1986). Development and validation of a scale for hypomanic personality. Journal of Abnormal Psychology, 95, 214–222. 10.1037/0021-843X.95.3.214 3745642

[cpp2391-bib-0018] First M. B., Spitzer R. L., Gibbon M., & Williams J. B. W. (Eds.) (1997). Structured clinical interview for DSM‐IV axis 1 disorders (Research ed.). New York: Biometrics Research.

[cpp2391-bib-0019] Fulford, D. , Johnson, S. , Llabre, M. , & Carver, C. (2010). Pushing and coasting in dynamic goal pursuit: Coasting is attenuated in bipolar disorder. Psychological Science, 21, 1021–1027. 10.1177/0956797610373372 20519486 PMC3162310

[cpp2391-bib-0021] Glickman, M. E. , Rao, S. R. , & Schultz, M. R. (2014). False discovery rate control is a recommended alternative to Bonferroni‐type adjustments in health studies. Journal of Clinical Epidemiology, 67, 850–857. 10.1016/j.jclinepi.2014.03.012 24831050

[cpp2391-bib-0022] Godoy‐Izquierdo, D. , Lo'pez‐Chicheri, I. , Lo'pez‐Torrecillas, F. , Ve'lez, M. , & Godoy, J. (2007). Contents of lay illness models dimensions for physical and mental diseases and implications for health professionals. Patient Education and Counseling, 67, 196–213. 10.1016/j.pec.2007.03.016 17462850

[cpp2391-bib-0023] Hagger, M. S. , & Orbell, S. (2003). A meta‐analytic review of the common‐sense model of illness representations. Psychology & Health, 18, 141–184. 10.1080/088704403100081321

[cpp2391-bib-0024] Havermans, R. , Nicolson, N. , Berkhof, J. , & deVries, M. (2010). Mood reactivity to daily events in patients with remitted bipolar disorder. Psychiatry Research, 179, 47–52. 10.1016/j.psychres.2009.10.020 20478632

[cpp2391-bib-0025] Havermans, R. , Nicolson, N. , Berkhof, J. , & deVries, M. (2011). Patterns of salivary cortisol secretion and responses to daily events in patients with remitted bipolar disorder. Psychoneuroendocrinology, 36, 258–265. 10.1016/j.psyneuen.2010.07.016 20732746

[cpp2391-bib-0026] Havermans, R. , Nicolson, N. , & Devries, M. (2007). Daily hassles, uplifts, and time use in individuals with bipolar disorder in remission. Journal of Nervous and Mental Disease, 195, 745–751. 10.1097/NMD.0b013e318142cbf0 17984774

[cpp2391-bib-0027] Healy, D. , & Williams, J. (1989). Moods, misattributions and mania: An interaction of biological and psychological factors in the pathogenesis of mania. Pyschiatric Developments, 7, 49–70. 2552433

[cpp2391-bib-0028] Higbed, L. , & Fox, J. (2010). Illness perceptions in anorexia nervosa: A qualitative investigation. British Journal of Clinical Psychology, 49, 307–325. 10.1348/014466509X454598 19580703

[cpp2391-bib-0030] Hochberg, Y. , & Benjamini, Y. (1990). More powerful procedures for multiple significance testing. Statistics in Medicine, 9, 811–818. 10.1002/sim.4780090710 2218183

[cpp2391-bib-0031] Jones, S. H. (2001). Circadian rhythms, multilevel models of emotion and bipolar disorder—An initial step towards integration? Clinical Psychology Review, 21, 1193–1209. 10.1016/S0272-7358(01)00111-8 11702512

[cpp2391-bib-0032] Jones, S. H. , & Day, C. (2008). Self appraisal and behavioural activation in the prediction of hypomanic personality and depressive symptoms. Personality and Individual Differences, 45, 643–648. 10.1016/j.paid.2008.07.008

[cpp2391-bib-0033] Jones, S. H. , Hare, D. J. , & Evershed, K. (2005). Actigraphic assessment of circadian activity and sleep patterns in bipolar disorder. Bipolar Disorders, 7, 176–186. 10.1111/j.1399-5618.2005.00187.x 15762859

[cpp2391-bib-0034] Jones, S. H. , Mansell, W. , & Waller, L. (2006). Appraisal of hypomania‐relevant experiences: Development of a questionnaire to assess positive self‐dispositional appraisals in bipolar and behavioural high risk samples. Journal of Affective Disorders, 93, 19–28. 10.1016/j.jad.2006.01.017 16503056

[cpp2391-bib-0035] Jones, S. H. , Smith, G. , Mulligan, L. D. , Lobban, F. , Law, H. , Dunn, G. , … Morrison, A. P. (2015). Recovery‐focused cognitive‐behavioural therapy for recent‐onset bipolar disorder: Randomised controlled pilot trial. British Journal of Psychiatry, 206, 58–66. 10.1192/bjp.bp.113.141259 25213157

[cpp2391-bib-0036] Judd, L. , Akiskal, H. , Schetteler, P. , Coryell, W. , Endicott, J. , Maser, J. , … Keller, M. (2003). A prospective investigation of the natural history of the long‐term weekly symptomatic status of bipolar II disorder. Archives of General Psychiatry, 60, 261–269. 10.1001/archpsyc.60.3.261 12622659

[cpp2391-bib-0037] Judd, L. L. , Akiskal, H. S. , Schetteler, P. J. , Endicott, J. , Maser, J. , Solomon, D. A. , … Keller, M. B. (2002). The long‐term natural history of the weekly symptomatic status of bipolar I disorder. Archives of General Psychiatry, 59, 530–537. 10.1001/archpsyc.59.6.530 12044195

[cpp2391-bib-0038] Knowles, R. , Tai, S. , Jones, S. , Highfield, J. , Morriss, R. , & Bentall, R. (2007). Stability of self‐esteem in bipolar disorder: Comparisons among remitted bipolar patients, remitted unipolar patients and healthy controls. Bipolar Disorders, 9, 490–495. 10.1111/j.1399-5618.2007.00457.x 17680919

[cpp2391-bib-0039] Kwapil, T. R. , Miller, M. B. , Zinser, M. C. , Chapman, L. J. , Chapman, J. , & Eckblad, M. (2000). A longitudinal study of high scorers on the Hypomanic Personality Scale. Journal of Abnormal Psychology, 109(2), 222–226. 10895560

[cpp2391-bib-0040] Leventhal, H. , Nerenz, D. , & Steele, D. (1984). Illness representations and coping with health threats In Singer B. (Ed.), A Handbook of Psychology and Health. Hillsdale: NJ: Erlbaum.

[cpp2391-bib-0041] Lobban, F. , Barrowclough, C. , & Jones, S. H. (2004). The impact of beliefs about mental health problems and coping on outcome in schizophrenia. Psychological Medicine, 34, 1165–1176. 10.1017/S003329170400203X 15697043

[cpp2391-bib-0042] Lobban, F. , Solis‐Trapala, I. , Tyler, E. , Chandler, C. , & Morriss, R. (2013). The role of beliefs about mood swings in determining outcome in bipolar disorder. Cognitive Therapy and Research, 37, 51–60. 10.1007/s10608-012-9452-9

[cpp2391-bib-0043] Lobban, F. , Taylor, K. , Murray, C. , & Jones, S. (2012). Bipolar disorder is a two‐edged sword: A qualitative study to understand the positive edge. Journal of Affective Disorders, 141, 204–212. 10.1016/j.jad.2012.03.001 22472729

[cpp2391-bib-0044] Mansell, W. (2006). The Hypomanic Attitudes and Positive Predictions Inventory (HAPPI): A pilot study to select cognitions that are elevated in individuals with bipolar disorder compared to non‐clinical controls. Behavioural and Cognitive Psychotherapy, 34, 467–476. 10.1017/S1352465806003109

[cpp2391-bib-0045] Mansell, W. , & Jones, S. H. (2006). The Brief‐HAPPI: A questionnaire to assess cognitions that distinguish between individuals with a diagnosis of bipolar disorder and non‐clinical controls. Journal of Affective Disorders, 93, 29–34. 10.1016/j.jad.2006.04.004 16697468

[cpp2391-bib-0046] Mansell, W. , Morrison, A. P. , Reid, G. , Lowens, I. , & Tai, S. (2007). The interpretation of, and responses to, changes in internal states: An integrative cognitive model of mood swings and bipolar disorders. Behavioural and Cognitive Psychotherapy, 35, 515–539. 10.1017/S1352465807003827

[cpp2391-bib-0047] Mansell, W. , Paszek, G. , Seal, K. , Pedley, R. , Jones, S. , Thomas, N. , … Dodd, A. (2011). Extreme appraisals of internal states in bipolar I disorder: A multiple control group study. Cognitive Therapy and Research, 35, 87–97. 10.1007/s10608-009-9287-1

[cpp2391-bib-0048] Mansell, W. , Rigby, Z. , Tai, S. , & Lowe, C. (2008). Do current beliefs predict hypomanic symptoms beyond personality style? Factor analysis of the Hypomanic Attitudes and Positive Predictions Inventory (HAPPI) and its association with hypomanic symptoms in a student population. Journal of Clinical Psychology, 64, 450–465. 10.1002/jclp.20455 18327768

[cpp2391-bib-0049] Miklowitz, D. , Goodwin, G. , Bauer, M. , & Geddes, J. (2008). Common and specific elements of psychological treatments for bipolar disorder: A survey of clinicians participating in randomized trials. Journal of Psychiatric Practice, 14, 77–85. 10.1097/01.pra.0000314314.94791.c9 18360193 PMC2603054

[cpp2391-bib-0050] Morrison, A.P. , Law, H. , Barrowclough, C. , Bentall, R.P. , Haddock, G. , Jones, S.H. , Kilbride, M. , Pitt, E. , Shryane, N. , Tarrier, N. , Welford, M. , & Dunn, G. (2016) Psychological approaches to understanding and promoting recovery in psychosis and bipolar disorder: A mixed‐methods approach. (UK): NIHR Journals Library (Programme Grants for Applied Reserch, 4.5). https://www.ncbi.nlm.nih.gov/books/NBK361044/ 27170958

[cpp2391-bib-0051] Myin‐Germeys, I. , Delespaul, P. , & van Os, J. (2003). The experience sampling method in psychosis research. Current Opinion in Psychiatry, 16, S33–S38. 10.1097/00001504-200304002-00006

[cpp2391-bib-0052] National Institute for Health and Clinical Excellence (NICE) (2014). Bipolar disorder: Assessment and management. The British Psychological Society and Gaskell.

[cpp2391-bib-0053] Oud, M. , Mayo‐Wilson, E. , Braidwood, R. , Schulte, P. , Jones, S. , Morriss, R. , … Kendall, T. (2016). Psychological interventions for adults with bipolar disorder: Systematic review and meta‐analysis. British Journal of Psychiatry, 208, 213–222. 10.1192/bjp.bp.114.157123 26932483

[cpp2391-bib-0054] Palmier‐Claus, J. , Myin‐Germeys, I. , Barkus, E. , Bentley, L. , Udachina, A. , Delespaul, P. , … Dunn, G. (2011). Experience sampling research in individuals with mental illness: Reflections and guidance. Acta Psychiatrica Scandinavica, 123, 12–20. 20712828 10.1111/j.1600-0447.2010.01596.x

[cpp2391-bib-0055] Pavlickova, H. , Varese, F. , Smith, A. , Myin‐Germeys, I. , Turnball, O. , Emsley, R. , & Bentall, R. (2013). The dynamics of mood and coping in bipolar disorder: Longitudinal investigations of the inter‐relationship between affect, self‐esteem and response styles. PLoS ONE, 8, e62514 10.1371/journal.pone.0062514 23638104 PMC3637453

[cpp2391-bib-0056] Peay, H. L. , Rosenstein, D. L. , & Biesecker, B. B. (2013). Adaptation to bipolar disorder and perceived risk to children: A survey of parents with bipolar disorder. BMC Psychiatry, 13, 327 10.1186/1471-244X-13-327 24294897 PMC3879194

[cpp2391-bib-0058] Power, M. J. , & Dalgleish, T. (1997). Cognition and emotion: From order to disorder. Hove and New York: Psyhcology Press.

[cpp2391-bib-0059] Proudfoot, J. , Parker, G. , Manicavasagar, V. , Hadzi‐Pavlovic, D. , Whitton, A. , Nicholas, J. , … Burckhardt, R. (2012). Effects of adjunctive peer support on perceptions of illness control and understanding in an online psychoeducation program for bipolar disorder: A randomised controlled trial. Journal of Affective Disorders, 142, 98–105. 10.1016/j.jad.2012.04.007 22858215

[cpp2391-bib-0060] R Development Core Team . (2011). R: A language and environment for statistical computing. Vienna, Austria.

[cpp2391-bib-0062] Watson, D. , Clark, L. A. , & Tellegen, A. (1988). Development and validation of brief measures of positive and negative affect: The PANAS scales. Journal of Personality and Social Psychology, 54, 1063–1070. 10.1037/0022-3514.54.6.1063 3397865

[cpp2391-bib-0063] Williams, J. , Kobak, K. , Bech, P. , Engelhardt, N. , Evans, K. , Lipsitz, J. , … Kalalie, A. (2008). The GRID‐HAMD: Standardization of the Hamilton Depression Rating Scale. International Clinical Psychopharmacology, 23, 120–129. 10.1097/YIC.0b013e3282f948f5 18408526

